# The Morphological Dependence of PEDOT on the Supporting Electrolytes Used and the Acquisition of Gold Nanoparticles with a View to Their Use in the Covalent Modification of the Ki-67 Antibody

**DOI:** 10.3390/polym17050672

**Published:** 2025-03-02

**Authors:** L. A. Hernández, I. D. M. Figueroa, G. Riveros, M. Luengo, E. Muñoz

**Affiliations:** 1 Facultad de Ciencias, Instituto de Química y Bioquímica, Universidad de Valparaíso, Valparaíso 2360102, Chile; isabeau.figueroa@alumnos.uv.cl (I.D.M.F.); gonzalo.riveros@uv.cl (G.R.); matias.luengo@postgrado.uv.cl (M.L.); 2 Facultad de Ciencias, Instituto de Química, Pontificia Universidad Católica de Valparaíso, Valparaíso 2373223, Chile; eduardo.munoz.c@pucv.cl

**Keywords:** conductive polymers, PEDOT, nanoparticles, electrosynthesis, gold, morphology, biosensors, Ki-67

## Abstract

We studied the influence of different supporting electrolytes (TBAPF_6_, TMAPF_6_, TEAPF_6_, TBAClO_4_, and LiClO_4_) on the morphology of PEDOT films electrochemically polymerized on screen-printed carbon electrodes, as part of which the synthesis of gold nanoparticles was tested for the subsequent modification of Ki-67 antibodies. Electrochemical deposition of the polymer was carried out using cyclic voltammetry and was characterized in the same way in solutions without the monomer. The nanoparticles were obtained using chronoamperometry at a constant potential for 3 s. The processes of p- and n-doping/undoping of both deposits (with and without gold) were studied, as was their characterization using SEM and ESEM-EDS. It was found that the supporting electrolytes intervened in the morphology and conductivity of the polymer films. In all films, it was possible to electrosynthesize gold nanoparticles, but the type of supporting electrolyte also influenced their distribution, showing that for this study, the most suitable were those obtained using TBAPF6, giving the most promising results for the covalent modification of antibodies to obtain future biosensors.

## 1. Introduction

In the last century, polymeric materials have revolutionized several sectors of industry, contributing significantly to technology, construction, food, and agriculture, among other areas of economic importance [1].

Among these materials, conductive polymers (CPs) stand out due to their particularity as organic macromolecules with properties belonging to inorganic semiconductor materials [2,3]. Since their discovery (1977), CPs have allowed us to project and think about new areas of application where they can be developed and enhance new technologies.

Among the most studied CPs, polypyrrole (PPy), polyaniline (PANI), and polythiophene (PTh), among others, stand out due to their conductivities [2]. Another striking feature of these materials is the versatility they present in their synthesis and doping, which can be carried out by chemical or electrochemical means, the latter through anodic oxidation of their respective monomers, leaving an oxidized polymer in situ during the process [4].

As stated, PTh is one of the most used CPs due to its conductive properties; however, the difficulty of its acquisition due to the need for anhydrous environments to polymerize it has directed attention towards a derivative, poly(3,4-ethylenedioxythiophene) (PEDOT), which has properties very similar to PTh but the advantage of being synthesizable under environmental conditions without the need for aprotic solvents or extreme conditions. In addition, its low oxidation potential, good environmental stability, and biocompatibility have positioned it for use in numerous commercial and research applications, such as capacitors, photovoltaic cells, photodiodes, and biosensors [5].

Despite it being a better option than other CPs, the growth, morphology, and electrical properties of the films of this polymer are susceptible and depend on the parameters and conditions applied during electrosynthesis [6,7].

In this way, the support electrolytes used, as well as the times and techniques according to which the electropolymerization of the films is carried out, must be chosen to obtain a polymer film that is best adapted to the use requirements. For example, the application of nanoparticles (NPs) of noble metals or metal oxides has become a widely studied technique in recent years, and its uses are primarily focused on the development of diagnostic devices such as biosensors, where antibodies, nucleotides, and DNA, among other biological molecules, bind to the nanoparticles already obtained on the electrode surfaces [8,9,10,11], a process in which the correct acquisition of a film of this polymer for its subsequent modification with NPs requires orderly and careful planning.

Thus, the objective of this article was to study the morphological differences in the PEDOT deposits obtained under the same electrosynthesis conditions when only modifying the supporting electrolytes—or counter ions—used during this process in order to then observe whether there was any difference in the acquisition of gold NPs. This was based on the fact that we know from previous studies that the salts that act as the supporting electrolyte can considerably influence the morphology of the CPs and their conductive properties and as such also influence the growth and distribution of metallic NPs in some way, with a view to their future use in electrodes for the development of biosensors intended for bio-detection. The salts used as the supporting electrolyte in this study were the following: tetramethylammonium hexafluorophosphate (TMAPF_6_), tetraethylammonium hexafluorophosphate (TEAPF_6_), lithium perchlorate (LiClO_4_), tetrabutylammonium hexafluorophosphate (TBAPF_6_), and tetrabutylammonium perchlorate (TBAClO_4_).

## 2. Materials and Methods

### 2.1. Materials

The monomer, 97%3,4-ethylenedioxythiophene (EDOT); the supporting electrolytes tetramethylammonium hexafluorophosphate (TMAPF_6_), 99%, tetraethylammonium hexafluorophosphate (TEAPF_6_), 99%, and lithium perchlorate (LiClO_4_), 95%; cysteamine, 95%; glutaraldehyde solution, grade II, 25% in H_2_O; and BSA were sourced from Sigma Aldrich, while the electrolytes tetrabutylammonium hexafluorophosphate (TBAPF_6_), 99%, and tetrabutylammonium perchlorate (TBAClO_4_), 98%, were sourced from AK Scientific (Union city, CA, USA) and Fluka analytics (Sigma Aldrich, St. Louis, USA), respectively.

The acetonitrile used as the solvent, CH_3_CN, 99.9%, was obtained from J. T. Baker (New Jersey, USA). Finally, 99.99% chloroauric acid (HAuCl_4_) and 99% potassium nitrate (KNO_3_) were obtained from AK Scientific and Genaxxon bioscience (Union city, CA, USA), respectively.

### 2.2. Methods and Instrumentation

All experiments were carried out at room temperature (20 °C) under an inert argon atmosphere in a three-compartment anchor-type glass cell. Screen-printed carbon electrodes (SPE) with a geometric area of 1 cm^2^ were used as the working electrode. A platinum (Pt) wire in the form of a spiral with a large geometric area was used as the auxiliary electrode, and an Ag|AgCl electrode was used as the reference electrode. All potentials presented in this article refer to this electrode.

The growth of poly 3,4-ethylenedioxythiophene (PEDOT) deposits was carried out by cyclic voltammetry between the potentials of −1.0 V and 1.5 V at a scan rate of 0.100 V s^−1^, in solutions containing 0.01 mol L^−1^ of EDOT and 0.1 mol L^−1^ of X in acetonitrile (CH_3_CN).

The electrochemical synthesis of gold nanoparticles is carried out in an anchor-type cell with identical conditions to those used for the synthesis of the polymer, using the PEDOT-modified electrode as the working electrode. For this purpose, a 6 mmol L^−1^ aqueous solution of HAuCl_4_ with 0.1 mol L^−1^ of KNO_3_ (supporting electrolyte). The reduction is carried out by chronoamperometry by applying a pulse of −0.50 V for 3 s. Finally, before modification with antibodies, the electrode must be cleaned with plenty of Milli-Q water to remove excess unreacted salt.

For the modification with the Ki-67 antibody, the electrode was incubated in a 50 mmol L^−1^ cysteamine solution (in milli Q water) for 1 h. The electrode was washed with a PBS solution (pH 7.3) for 15 min and then incubated in a 2.5% glutaraldehyde solution in PBS for 45 min. After this time, it was washed again for 15 min in a PBS solution (pH of 7.3). The electrode was then incubated with the Ki-67 antibody at a concentration of 10 μg/mL for 1 h. It was then washed again with PBS for 15 min.

All electrochemical measurements were carried out with a CH Instruments CHI660D model potentiostat (Austin, TX, USA).

Finally, the equipment used for the morphological characterization of the samples was the Field Emission Scanning Electron Microscope ESEM Quattro S (Thermo Fischer Scientific, Waltham, MA, USA).

## 3. Results and Discussion

### 3.1. Electropolymerization of PEDOT: Influence of Salts on Voltammetric Profiles

For the electrochemical synthesis of PEDOT on ITO, the parameters present in the literature were used, as studied by the group of del Valle et al. (2015) [12], who placed an appropriate window for the synthesis of the polymer by electro oxidation by cyclic voltammetry (CVs) between −1.0 V and 1.5 V, according to which PEDOT deposits were made in 3 cycles.

**Figure 1:**
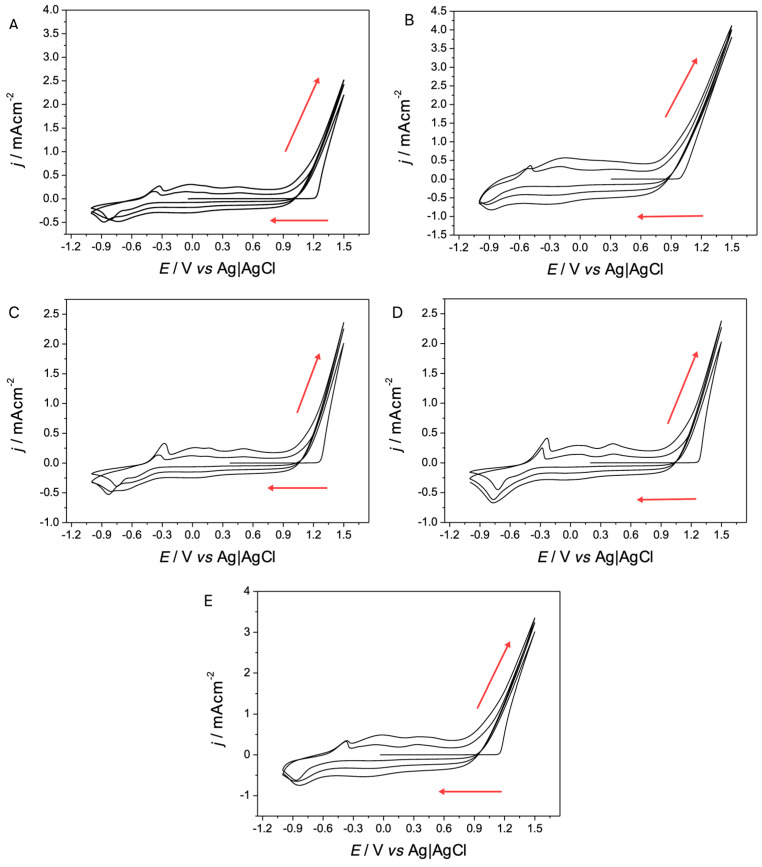
Cyclic voltammograms of the preparation of PEDOT films with the different supporting electrolytes in a potential window between −1.0 and 1.5 V. Interface: SPE + 0.01 mol L^−1^ of EDOT and 0.1 mol L^−1^ X in CH_3_CN, v = 100 mV/s, 3 cycles. Where X is (**A**) TBAPF_6_, (**B**) TMAPF_6_, (**C**) TEAPF_6_, (**D**) TBAClO_4_ y, (**E**) LiClO_4_. (red arrow: electrochemical oxidation).

The voltammograms obtained (Figure 1) have a profile like those reported in the literature, as well as the coloration of the deposits obtained (dark blue) [12]. Furthermore, all the profiles in global form show the exponential growth of the current response from 0.9 V, observing more conductive profiles as the cycles increase, which is indicative of obtaining PEDOT [13,14,15].

A more detailed analysis between voltammograms, comparing the different salts used as a supporting electrolyte, allows the influence of the salt used in its electropolymerization to be observed. This begins from the basis that the obtaining conditions in each film are the same, and only the volume of the salt used varies (TBAPF_6_ 300.36 Å^3^, TMAPF_6_ 71.35 Å^3^, TEAPF_6_ 164.34 Å^3^, TBAClO_4_ 300.36 Å^3^, and LiClO_4_ 57.09 Å^3^) (https://chemicalize.com/ (accessed on 16 December 2024)). In fact, EDOT solutions containing smaller cations such as TMA^+^ and Li^+^ show faster oligomer formation processes, reaching higher anodic currents, than those with larger counterions, as is the case in Figure 1A,C,D which correspond to TBA^+^, TEA^+^, TBA^+^, respectively. These differences in the profiles could correspond to some blocking effect that the larger cations could be produced at the charged electrode–solution interface, generating a lower rate of active species that initiate electropolymerization.

### 3.2. Effect of Supporting Electrolytes on the Morphology of PEDOT Films

**Figure 2:**
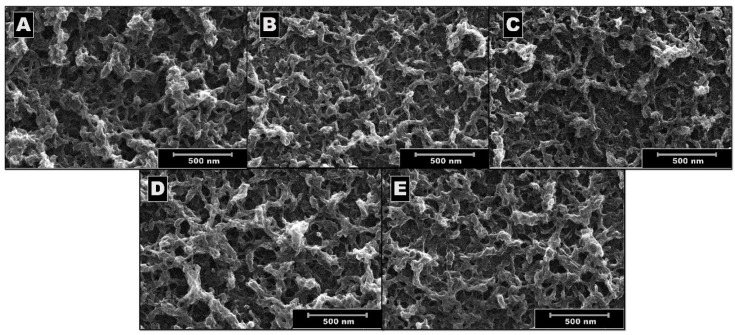
SEM images at 500 nm of PEDOT deposits on ITO electrosynthesized by VC for 3 cycles at a scan rate of 100 mV/s between −1.0 and 1.5 V with different supporting electrolytes, (**A**) TBAPF_6_, (**B**) TMAPF_6_, (**C**) TEAPF_6_, (**D**) TBAClO_4_, and (**E**) LiClO_4_.

Figure 2 shows the images of the SEM morphological characterization of the five PEDOT deposits. Corroborating the observations in the previous section, micrographs 2A, C and D, corresponding to the films deposited using TBAPF_6_, TEAPF_6_, and TBAClO_4_ deposits, respectively, have a structure with more open spaces or cavities, showing greater porosity, which could be due to the size of the counterions involved in electrosynthesis. Likewise, the morphologies obtained using LiClO_4_ (Figure 2E) and TMAPF_6_ (Figure 2B) show more closed cavities, indicating more homogeneous and compact surfaces, which could influence future modifications that may be made to the PEDOT films.

From these micrographs and relating them to the observed electropolymerization profiles, it could be inferred that when using electrolytes with size differences of up to 5 times, for example, between LiClO_4_ and TBAPF_6_, the nucleation and growth mechanisms [13,14] seem to occur in different kinetics (faster nucleation in the case of smaller ions), which would mean that per area, a greater number of polymeric growth points would arise when the ions are smaller, describing more compact or less rough morphologies.

### 3.3. Voltammetric Responses of PEDOT Films

**Figure 3:**
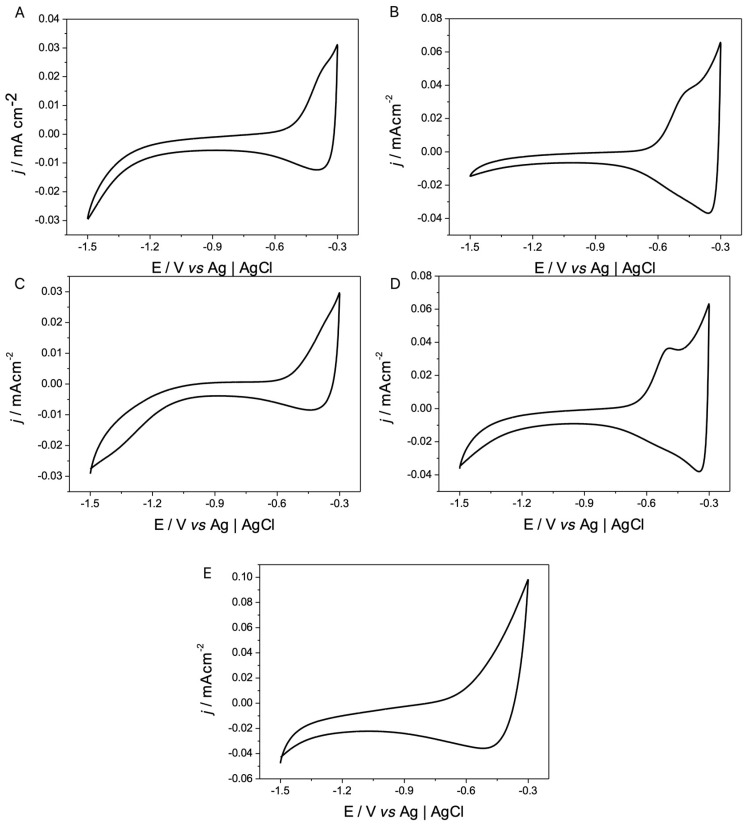
Cyclic voltammograms of n-doping/undoping processes of the PEDOT-coated electrodes from Figure 1. Interface: PEDOT + 0.1 mol L^−1^ X in CH_3_CN, ν = 50 mv/s, where X is (**A**) TBAPF_6_, (**B**) TMAPF_6_, (**C**) TEAPF_6_, (**D**) TBAClO_4_, and (**E**) LiClO_4_.

**Figure 4:**
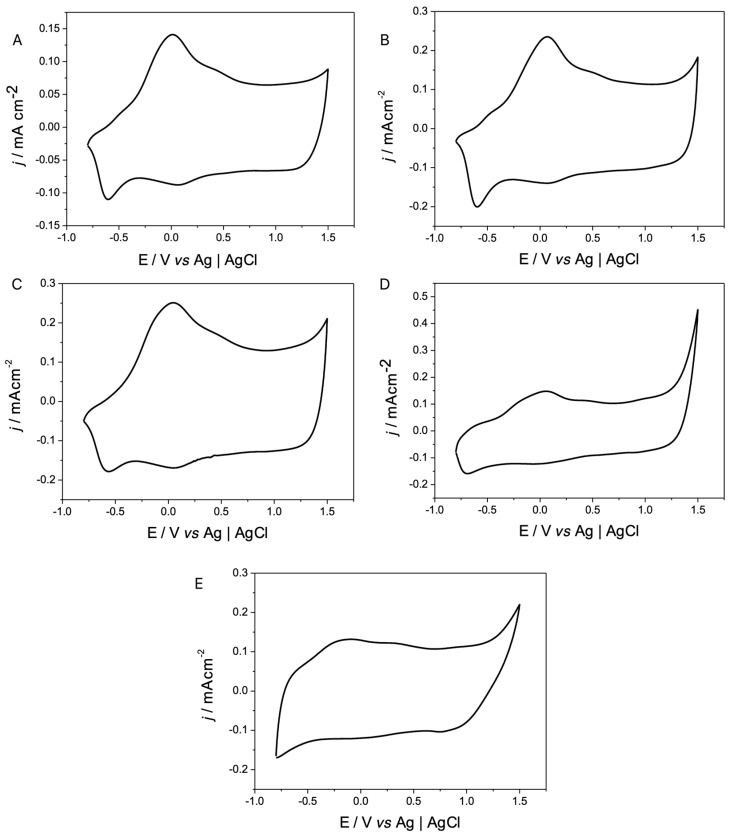
Cyclic voltammograms of the p-doping/undoping processes of the PEDOT-coated electrodes in Figure 1. Interface: PEDOT + 0.1 mol L^−1^ X in CH_3_CN, v = 50 mv/s, where X is: (**A**) TBAPF_6_, (**B**) TMAPF_6_, (**C**) TEAPF_6_, (**D**) TBAClO_4_, and (**E**) LiClO_4_.

From the voltammetric profiles and micrographs obtained, it could be inferred that the use of different electropolymerization stages could be changing the electrical nature of the film. A study that allows the corroboration or discarding of this is the analysis of the voltammetric response of each deposit to subsequently separate and analyze in detail the p- and n-doping/undoping processes for PEDOT, which are presented in Figure 3 and Figure 4.

From these profiles, the charge corresponding to each of the processes was obtained to estimate their reversibility; these values are presented in Table 1. It is possible to infer from this table that both types of processes are electrochemically irreversible, but that the ratio between the p-doping/undoping process is closer to 1, indicating greater electrochemical reversibility.

In the case of the irreversibility of the n-doping/undoping process, this is because the values associated with the undoping charge (Q_n und_) are greater than those associated with doping (Q_n dop_).

From the results presented in Table 1, it can be observed that despite the differences in the morphologies obtained, due to the salts used, the doping and undoping processes that are so characteristic of PEDOT are not influenced by the degree of porosity that the films present, obtaining similar values in both processes to those reported in the literature for PEDOT.

### 3.4. Electrosynthesis of Gold Nanoparticles on PEDOT Films

To determine whether the modulation of different PEDOT morphologies due to the use of different supporting electrolytes would change the synthesis of gold NPs on them, the electrowinning of gold NPs on the obtained PEDOT deposits was tested.

**Figure 5:**
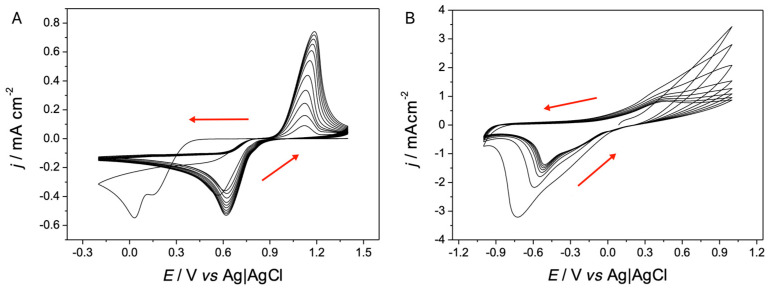
Cyclic voltammograms for electrochemical synthesis Au NPs on SPE (**A**) and PEDOT (**B**) in a potential window between − 0.2 and 1.4 V and − 1.1 and 1.0 V, respectively. Interface (**A**): SPE + 6 mmol L^−1^ of HAuCl_4_ with 0.1 mol L^−1^ of KNO_3_ in H_2_O, at ν = 0.05 Vs^−1^, 12 cycles. Interface (**B**): SPE|PEDOT + 6 mmol L^−1^ of HAuCl_4_ with 0.1 mol L^−1^ of KNO_3_ in H_2_O, at V = 0.05 V s^−1^, 8 cycles. (red arrow: electrochemical oxidation).

The electrochemical synthesis of Au on the SPE electrodes and on the PEDOT-modified electrodes shows an evident shift in the obtaining windows, exemplified in Figure 5, where a change in the conductive surface from −0.2 V and 1.4 V for the electrode without deposit to −1.1 and 1.0 V can be observed when obtained on the PEDOT using, for example, TBAPF_6_ as the supporting electrolyte.

The voltammetric profiles obtained for Figure 5A, B are similar to those reported in the literature [16]. In fact, from the voltammograms obtained for Figure 5A, a first reduction peak around 0.03 V can be observed, attributable to the reduction of Au (III) to Au (0) over ITO [16,17,18], in relation to the reports in the literature according to the following equations.

(1) $$HAuCl_{4}+3e^{-}\to Au+{4Cl}^{-}+H^{+}$$

Around 1.18 V, the anodic peaks are observed due to the oxidation of the gold deposited in the first cycle, according to the following equation:

(2) $$Au+Cl^{-}\to {\left[AuCl_{4}\right]}^{-}+3e^{-}$$

From the second cycle, it can be observed that the gold reduction peaks from Au (III) to Au (0) change their potential range between 0.61 and 0.62 V, which could indicate that the reduction of Au (III) to Au (0) begins to occur on the previously formed Au (0) nuclei, which would imply a lower energy expenditure [16,17].

In a very similar way to what was described above, Figure 5B shows a first reduction peak around −0.73 V and as in the previous case after the second cycle, the reductions of Au (III) to Au (0) present a potential shift and occur between −0.49 and −0.50 V.

With the data provided by these analyses, it was possible to optimize an adequate potential to obtain the gold NPs at short pulses on PEDOT, applying a potential of −0.50 V for 3 s for all the PEDOT deposits with the different electrolytes studied.

### 3.5. Characterization of Au-PEDOT NPs: Influence of the Supporting Electrolyte

**Figure 6:**
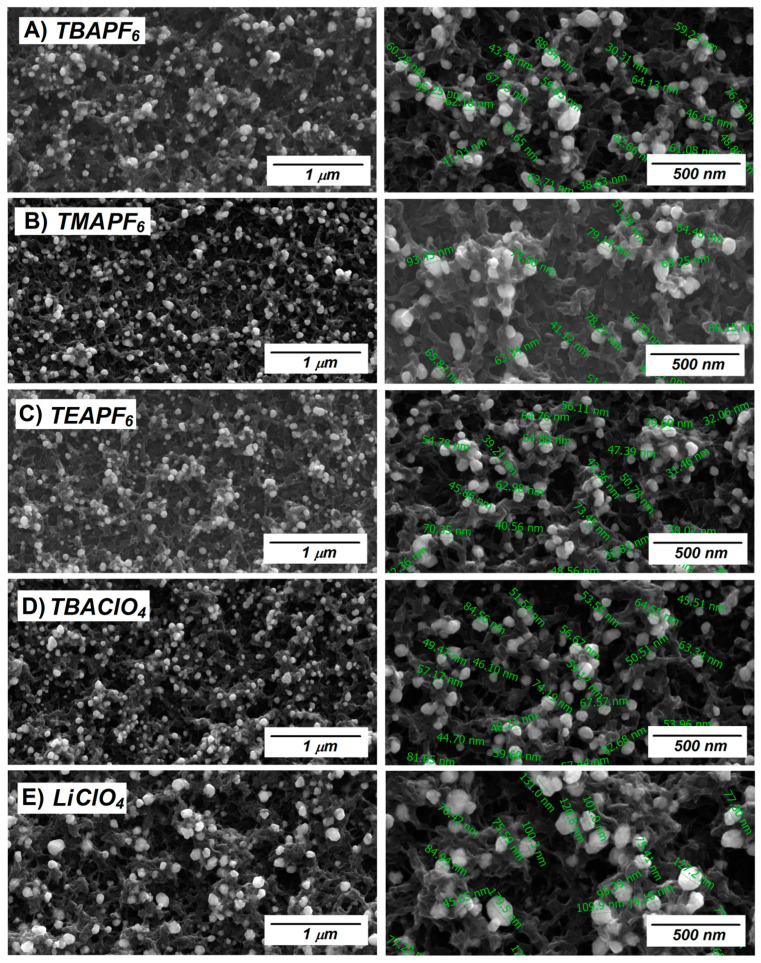
SEM micrographs at 1 um and 500 nm of the PEDOT deposits on ITO. (**A**) TBAPF_6_, (**B**) TMAPF_6_, (**C**) TEAPF6, (**D**) TBAClO_4_, and (**E**) LiClO_4_.

The gold deposits on the electrodes were characterized by SEM and can be seen in Figure 6.

In the micrographs presented, it is possible to see in these images that the controlled reduction of Au (III) to Au (0) on PEDOT-generated NPs that were smaller than 100 nm in all the electrodes observed. When analyzing each case in particular, a trend can be observed, already seen in the previous sections. In this case, the Au (0) deposits on PEDOT-TMAPF_6_ and PEDOT-LiClO_4_ (Figure 6B and Figure 6D, respectively), present the largest sizes observed, between 60 and 80 nm in the case of PEDOT-TMAPF_6_ and between 90 and 130 nm for PEDOT-LiClO_4_ (in both cases, these values represent a representative average). This is coincident with the morphologies of the less rough and more compact deposits, which could limit the increase in nucleation sites for a greater dispersion of NPs, which would increase the probability of aggregation between the NPs and by coincidence, also the largest sizes reported. For the other Au (0) deposits on PEDOT (Figure 6A,C,D), no major differences are observed between the sizes obtained, being located for these three cases between 20 and 70 nm approximately. This is again coincidental with the fact that the larger electrolyte sizes generated rougher and less compact PEDOT films, which ultimately translates into a greater number of nucleation sites during the Au salt reduction process, a greater Np distribution, and smaller and more homogeneous sizes [19].

In turn, considering that morphology can be related to conductivity, a less rough polymer with a greater crystalline character should exhibit higher conductivities than those that have fewer compact morphologies, as shown in Figure 2. Regarding this, studies carried out by del Valle et al. [19] on the influence of PEDOT conductivity, depending on the supporting electrolyte used, showed a significant trend from which they concluded that the conductivity can be ordered based on the doping anion of the supporting electrolyte used for the electropolymerization, whose sequence from highest to lowest conductivity would be ${PF}_{6}^{-}$ > ${ClO}_{4}^{-}$. The study by del Valle et al. [12] as well as other similar ones used CV as a starting technique to obtain the deposits. However, in this study, the morphology of the films obtained, and the NPs generated, have a direct trend, more with the size of the cation than the anion, with the only difference with the other reported studies being the electrochemical synthesis technique, and in this case, chronoamperometry.

On the other hand, if we analyze the techniques for obtaining the conductive polymer, we know that CV electrosynthesis is a slower technique, which gives time to the first oligomers that saturate the high oligomeric density region to precipitate on the electrode in a more orderly manner than could be obtained by chronoamperometry. However, an analysis that is not performed when changing the technique is how in the high oligomeric density region, the supporting electrolyte that is found in proportions of at least 10 times greater than the monomer concentration behaves in the vicinity of the electrode. Possibly, the phenomenon observed in this work can be explained by the kinetics of the polymerization processes; in fact, using chronoamperometry as the technique, it is possible to obtain a greater amount of polymeric deposit in a short time, representing a difference from what CV can achieve. In our case, it also seems that by increasing the kinetics of the electropolymerization reaction and by generating faster and higher proportion cation-radicals, which need counterions to obtain electroneutrality, they could in turn be increasing the concentration of cations in the vicinity of the electrode, conditioning the morphology. In addition, contrary to what was observed when using CV, the smaller cations generate less rough films or with more compact cavities in the case of PEDOT.

### 3.6. ESEM-EDS Analysis

**Figure 7:**
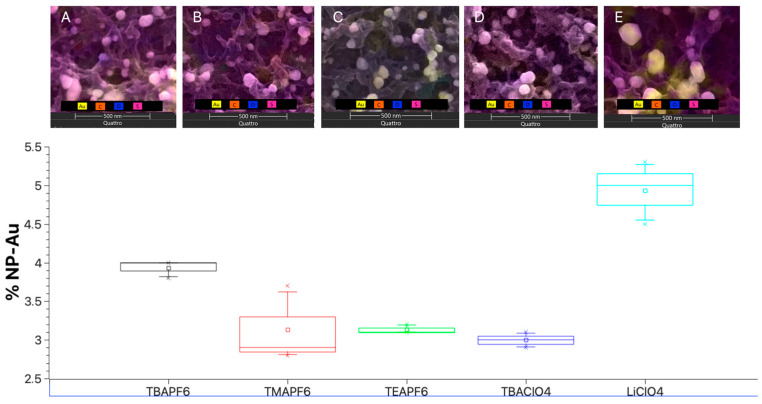
ESEM-EDS micrographs at 500 nm of PEDOT deposits on ITO. (**A**) TBAPF_6_, (**B**) TMAPF_6_, (**C**) TEAPF_6_, (**D**) TBAClO_4_, and (**E**) LiClO_4_, and their respective percentage distribution of gold on the conductive surface.

The analyses performed on the modified surfaces showed that the samples with TMAPF_6_ and LiClO_4_ have Au % distributions as nanoparticles with lower reproducibility between the samples than those obtained with the other supporting electrolytes, where it is possible (Figure 7) to observe samples that are more homogeneous in terms of the % of total Au distributed in the modified electrodes. From here, it is possible to infer that the supporting electrolytes not only modulate the nucleation and growth mechanisms for the polymer but also have an impact on the reproducibility between modifications, for example with gold, which is essential when using these as a platform for bioanalytical measurements, such as biosensors.

### 3.7. PEDOT-AuNPs Doping-Undoping

After the results observed by SEM and ESEM-EDS, it was analyzed whether the processes of p- and n-doping/undoping of PEDOT were altered by the sizes and dispersion of the Au NPs obtained influenced by the morphology of PEDOT.

**Figure 8:**
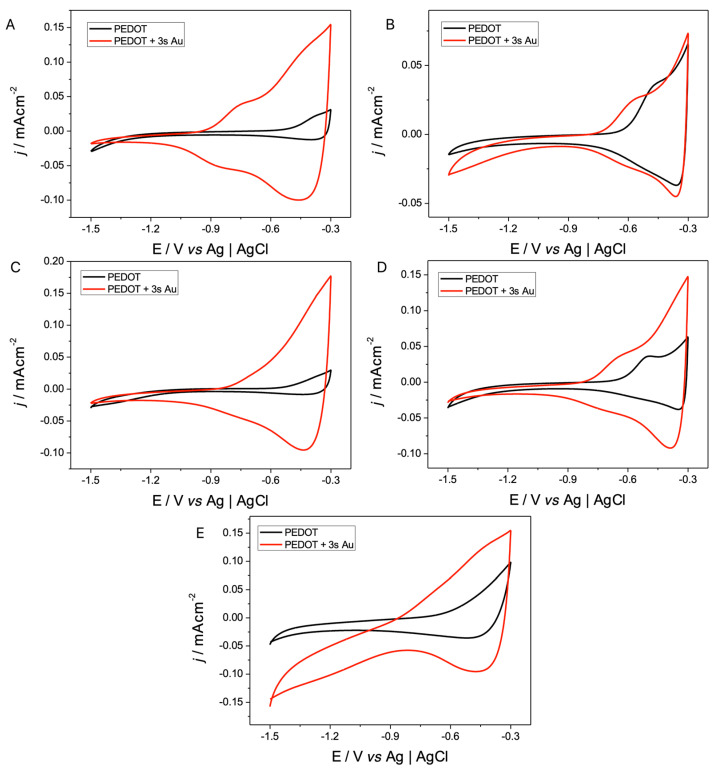
Cyclic voltammograms of n-doping/undoping processes of PEDOT-coated electrodes (black line) and PEDOT NPs Au-coated electrodes (red line). Interface: PEDOT + 0.1 mol L^−1^ X in CH_3_CN and PEDOT + 3 s NPs Au + 0.1 mol L^−1^ X in CH_3_CN, ν = 50 mV s^−1^, where X is (**A**) TBAPF_6_, (**B**) TMAPF_6_, (**C**) TEAPF_6_, (**D**) TBAClO_4_, and (**E**) LiClO_4_.

**Figure 9:**
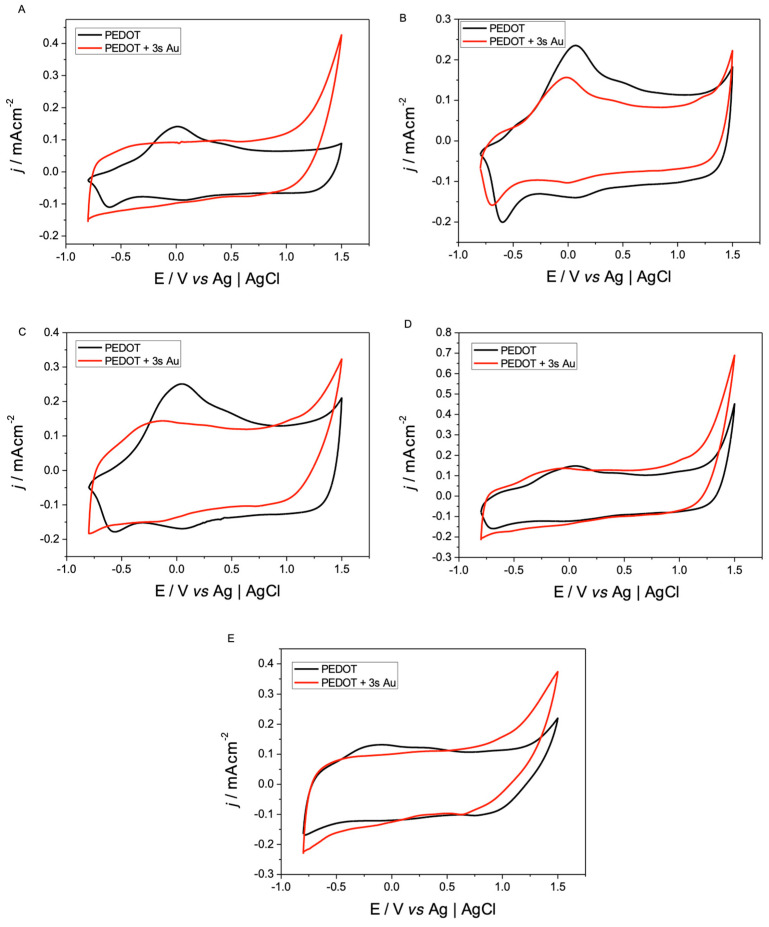
Cyclic voltammograms of the p-doping/undoping processes of the electrodes coated with PEDOT (black line) and PEDOT + Au NPs (red line). Interface: PEDOT + 0.1 mol L^−1^ X in CH_3_CN and PEDOT + 3 s Au NPs + 0.1 mol L^−1^ X in CH_3_CN ν= 50 mV s^−1^, where X is (**A**) TBAPF_6_, (**B**) TMAPF_6_, (**C**) TEAPF_6_, (**D**) TBAClO_4_, and (**E**) LiClO_4_.

The p- and n-doping/undoping processes presented in Figure 8 and Figure 9 were observed, and the ratios between the values of their charges are shown in Table 2. It can be seen that with the presence of Au NPs in the electrodes, the p-doping/undoping process does not vary considerably. The same is the case in the n-doping/undoping processes where, in addition to observing a considerable increase in the electrochemical response of the modified polymer, it is also possible to see that the ratio between the charge and discharge is closer to 1, compared to those reported in Table 1 for the process without the Au NPs present. This increase in the responses can positively influence the measurements of biosensors, increasing, for example, their sensitivity to analytes, decreasing the detection limits when working with electrodes with a greater reversibility when transporting ions from the heart of the solution and releasing them, when external measuring cups are used in amperometry and impedance techniques.

### 3.8. Immobilization of Ki-67 Antibodies

**Figure 10:**
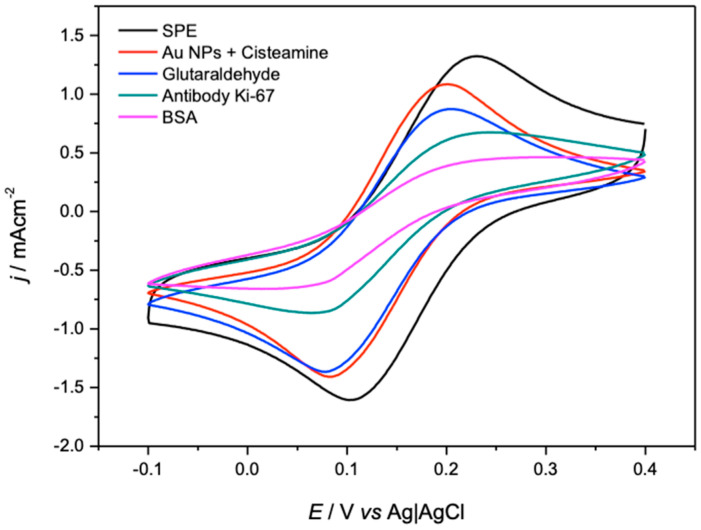
Electrochemical responses at each modification step with the Ki-67 antibody.

Finally, covalent immobilization tests were performed on the obtained nanoparticles. The hypothesis raised at the beginning of this study is that obtaining homogeneous distributions in size and quantity of NP-Au on the modified electrodes should improve the responses of the tested antibodies. Figure 10 shows the electrochemical responses obtained with an external ferrocene redox couple, which is used as a measure of how the surface of the electrodes is modified in each immobilization step. In this study, the best electrochemical response obtained was provided by the TBAPF_6_ salt, where we can observe the expected behavior in each modification step (progressive decrease in the intensity of the redox couple), with the salt giving the highest current response in the final step of the modification before being tested as a biosensor. In this section, the best response obtained is shown, which is with the TBAPF_6_ salt, which gives us high current responses despite the modification with biorecognition molecules that are characterized by blocking the active and conductive sites of the electrode, passivating it. However, it is possible to observe how the homogeneous distribution of the Au Np allows considerable current responses to be maintained after the covalent union of the antibodies for biosensors. (The other electrochemical responses can be visualized in the Supplementary Materials S1).

## 4. Conclusions

This study of the use of different supporting electrolytes for the electrochemical polymerization of PEDOT by cyclic voltammetry indicated that these effectively influence the surface morphology of the polymer film obtained, in particular, the size of the deposits regulates the roughness and the type of cavities or morphology of these deposits, so their choice should be related to the use required of them.

To discover whether the morphologies obtained by the use of different deposits would affect the deposits of metal NPs on the electrode, gold nanoparticles were deposited on them. In all electrodes, gold NPs were obtained, which presented spherical, non-perfect morphologies and similar sizes, except for the film obtained using LiClO_4_ where larger sizes were obtained; meanwhile, on the electrode made with TMAPF6, the NPs obtained were more delimited from each other, without forming agglomerations, which is related to the conductivity of this deposit.

Furthermore, when studying the p- and n-doping/undoping processes of the electrodes with and without Au NPs, these showed greater reversibility for the p-doping/undoping process, in both cases, and a notable irreversibility for the n doping/undoping process, which decreased somewhat for those electrodes that had Au NPs.

When testing the modification with the Ki-67 antibody, the electrode that shows more homogeneous and better distributed nanoparticle sizes (TBAPF6) allows covalent modifications to be obtained that presented high current responses (using an ferrocene redox couple), allowing us to conclude that the choice of the supporting electrolyte cannot be random and must be chosen based on systematic studies for the design of the most suitable material for biosensors.

It should be noted that in the future it would be valuable to be able to determine the conductivities of all the films made, as well as to test obtaining thinner and more homogeneous polymeric films with NPs that have a greater order in their surface arrangement with a view to future use as bases for the development of possible biosensors of interest in the health and agricultural industries.

## Figures and Tables

**Table 1:** Charges of the p- and n-doping/undoping processes (Q_p dop_/Q_p und_ and Q_n dop_/Q_n und_, respectively) obtained from the cycle 3 response of the PEDOT-modified electrodes in monomer-free solutions, each with its respective salt.

Samples	Q_p dop_ [C]	Q_p und_ [C]	Q_p dop_/Q_p und_	Q_n dop_ [C]	Q_n und_ [C]	Q_n dop_/Q_n und_
TBAPF_6_	0.00327	0.0033	1.00826	1.59774 × 10^−4^	2.31697 × 10^−4^	0.68958
TMAPF_6_	0.0056	0.00533	1.04972	2.61805 × 10^−4^	3.33513 × 10^−4^	0.78499
TEAPF_6_	0.00647	0.00621	1.04179	1.56328 × 10^−4^	2.22881 × 10^−4^	0.7014
TBAClO_4_	0.00537	0.00491	1.09297	3.18297 × 10^−4^	4.3059 × 10^−4^	0.73921
LiClO_4_	0.00522	0.00489	1.06655	4.60095 × 10^−4^	6.73326 × 10^−4^	0.68332

**Table 2:** Charges of the p- and n-doping/undoping process (Q_p dop_/Q_p und_ and Q_n dop_/Q_n und_, respectively) obtained from cycle 5 CV response of the electrodes modified with PEDOT and Au NPs in monomer-free solutions, each with its respective salt.

Samples	Q_p dop_ [C]	Q_p und_ [C]	Q_p dop_/Q_p und_	Q_n dop_ [C]	Q_n und_ [C]	Q_n dop_/Q_n und_
TBAPF_6_	0.00537	0.00457	1.17626	9.45064 × 10^−4^	4.72532 × 10^−5^	0.83621
TMAPF_6_	0.0056	0.00533	1.04972	2.61805 × 10^−4^	3.33513 × 10^−4^	0.78499
TEAPF_6_	0.0062	0.00556	1.11545	8.23916 × 10^−4^	9.84578 × 10^−4^	0.83682
TBAClO_4_	0.00742	0.0063	1.17819	6.9976 × 10^−4^	8.56955 × 10^−4^	0.81657
LiClO_4_	0.0061	0.00556	1.08461	0.00156	0.00207	0.75121

## Data Availability

All data can be obtained directly from the email: loreto.hernandez@uv.cl.

## Supplementary Materials

The following supporting information can be downloaded at: https://www.mdpi.com/article/10.3390/polym17050672/s1.
