# Supplementary figures and images for: The Morphological Dependence of PEDOT on the Supporting Electrolytes Used and the Acquisition of Gold Nanoparticles with a View to Their Use in the Covalent Modification of the Ki-67 Antibody
Source: Polymers (Basel). 2025 Mar 2;17(5):672. doi: 10.3390/polym17050672 (PMC11902629; doi:10.3390/polym17050672)

## Supplementary Material

### 1) TMAPF6.

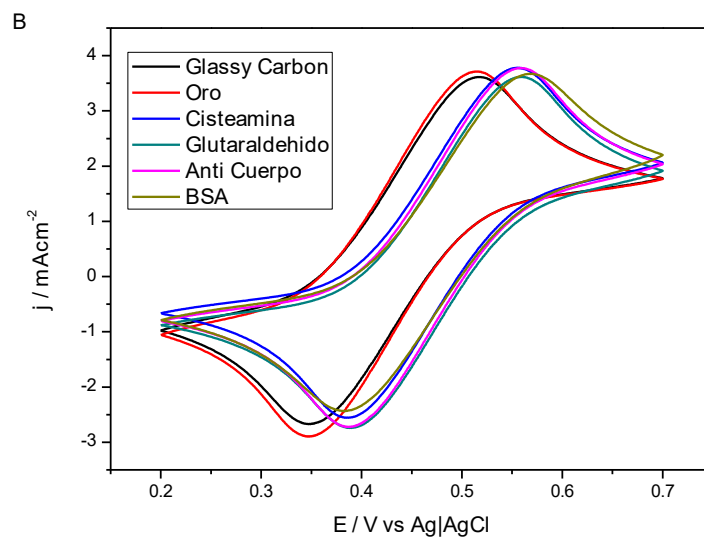

### 2) LiClO4

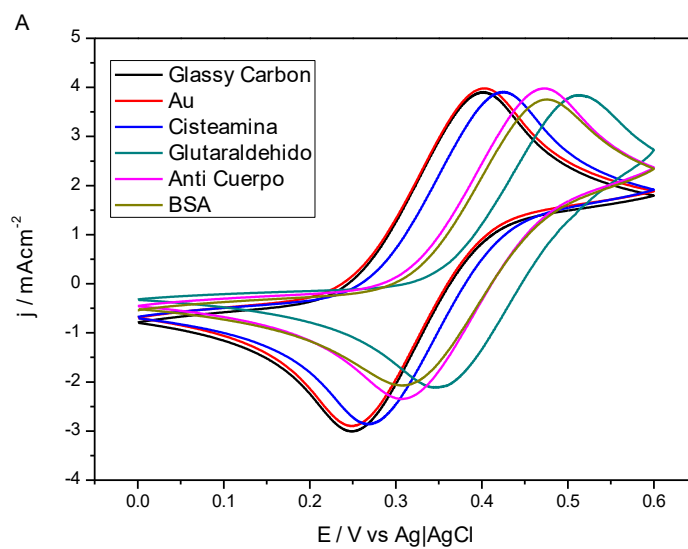

### 3) TEAPF6

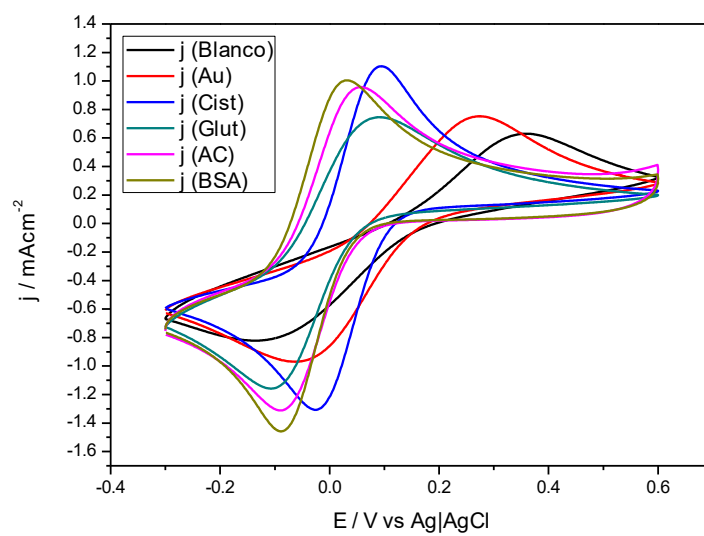

### 4) TBAClO4

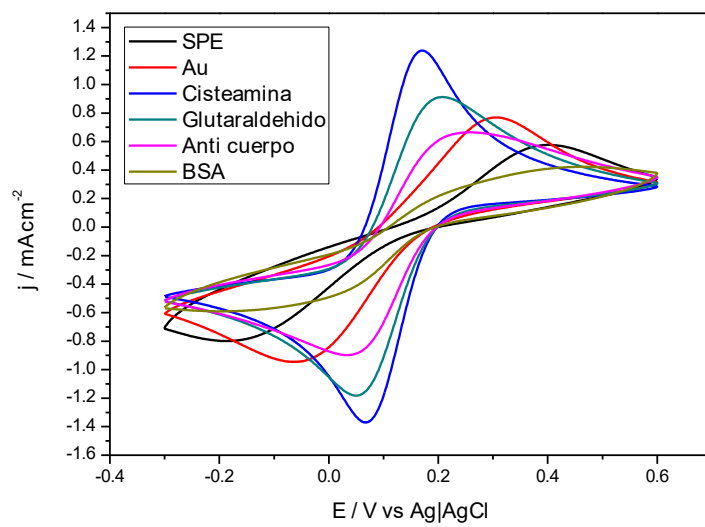

Supplement: Supplementary file 1 [file polymers-17-00672-s001.zip › polymers-3450714-supplementary.pdf]
